# Transcriptional Activation of the *TREM2* Gene by ZEB2 in a Zinc Finger-Dependent Manner

**DOI:** 10.3390/genes16111329

**Published:** 2025-11-03

**Authors:** Motoaki Yanaizu, Yuji Takata, Masahide Kato, Haruka Fujiwara, Yoshihiro Kino

**Affiliations:** Department of RNA Pathobiology and Therapeutics, Meiji Pharmaceutical University, 2-522-1, Noshio, Kiyose-shi, Tokyo 204-8588, Japan; m-yana@my-pharm.ac.jp (M.Y.);

**Keywords:** TREM2, transcription factor, ZEB2, Alzheimer’s disease

## Abstract

**Background/Objectives**: TREM2 is a transmembrane receptor highly expressed in microglia and macrophages, and its involvement in Alzheimer’s disease, obesity, and cancer has garnered significant attention. Although its biological function has been actively investigated, the mechanisms by which its expression is regulated remain incompletely characterized. In this study, we aimed to identify transcription factors that modulate TREM2 expression among those reported to be expressed in microglia. **Methods**: We inserted a 5 kb upstream region of *TREM2* into a luciferase reporter vector. This construct was co-expressed with 15 transcription factors, and the *TREM2* transcriptional activity was evaluated using luciferase assays. The most promising transcription factor was subsequently knocked down in HMC3 cells, which are derived from human microglia, to assess its effect on endogenous *TREM2* expression. **Results**: Among the 15 transcription factor candidates tested, SPI1 (PU.1), MAFB, CEBPA, ZEB2, and SALL1 most strongly enhanced *TREM2* transcriptional activity. ZEB2 was prioritized due to its limited study in microglia and higher co-expression with *TREM2*. In HMC3 cells, ZEB2 knockdown reduced both *TREM2* mRNA and protein levels. Further analysis using domain-deleted mutants of ZEB2 indicated that the zinc finger domains are essential for its transcriptional activity. Analysis using truncated mutants of the *TREM2* upstream region suggests that ZEB2 acts on multiple sites within this region. Chromatin immunoprecipitation also suggested an interaction between ZEB2 and the upstream region of *TREM2*. **Conclusions**: This study novelly suggests ZEB2 as a transcription factor that promotes TREM2 expression. Further investigation into the role of ZEB2 in various TREM2-associated diseases is warranted.

## 1. Introduction

Triggering receptor expressed on myeloid cells 2 (TREM2) is a type I transmembrane receptor protein expressed on immune cells such as microglia [1], macrophages [2], osteoclasts [3], and dendritic cells [4]. On the cell membrane, TREM2 forms a complex with the signaling adaptor molecule TYRO protein tyrosine kinase-binding protein (TYROBP) [5,6], functioning as a receptor protein on microglia in the brain. TREM2 possesses an extracellular IgV domain, which mediates ligand binding. Known ligands of TREM2 include proteins such as amyloid-β (Aβ) [7,8,9] and apolipoprotein E (ApoE) [10,11], as well as phospholipids such as phosphatidylserine [12]. Upon ligand engagement at the IgV domain, downstream signaling is transduced via TYROBP, leading to the activation of microglial proliferation and phagocytic activity [13].

Loss-of-function mutations in *TREM2* have long been recognized as the causative factor in Nasu–Hakola disease, a rare hereditary disorder characterized by pathological fractures due to polycystic bone lesions and early-onset dementia associated with leukoencephalopathy [14,15]. Subsequently, rare variants located within exon 2 of *TREM2*, including rs75932628 (R47H) and rs143332484 (R62H), have been reported to increase the risk of developing Alzheimer’s disease (AD) [16,17].

Alzheimer’s disease (AD) is a progressive neurodegenerative disorder characterized by cognitive decline. Its pathological hallmarks include the formation of extracellular senile plaques composed primarily of Aβ and the presence of neurofibrillary tangles—intracellular inclusions formed by hyperphosphorylated tau protein [18,19,20]. The aggregation and accumulation of Aβ are closely implicated in the onset and progression of AD [18].

The clearance of amyloid-beta is mediated by microglia, the cells responsible for immune surveillance within the brain. Microglia are a type of glial cell in the central nervous system that exhibit diverse functions adapted to the brain’s microenvironment, such as the phagocytosis of foreign substances [21,22], cytokine release [23], and synaptic pruning for neural circuit modulation [24]. TREM2 regulates these microglial functions. In the brains of patients with AD, microglia accumulate around Aβ plaques, suppress their spread, and mediate Aβ phagocytosis through TREM2 [25]. Microglia lacking *TREM2* or harboring the R47H variant of *TREM2* fail to accumulate near Aβ plaques [25,26].

Comprehensive transcriptomic analyses have revealed that microglia represent a heterogeneous population whose phenotypic states dynamically shift in response to environmental changes [27,28,29]. A distinct subset known as disease-associated microglia (DAM)—characterized by specific transcriptional profiles—has been identified in both AD model mice and AD patients. DAMs emerge in the context of aging and AD progression and display phagocytic and proinflammatory activity [27]. While DAMs may attenuate neurodegeneration in certain mouse models, inappropriate DAM activation can exacerbate neurodegenerative pathology [30].

The transition from homeostatic microglia to DAMs involves a two-step activation process, during which the expression levels of numerous genes fluctuate. *TREM2* is among the genes upregulated during the second step of DAM induction [27]. Impaired progression to the second phase of DAM differentiation in *Trem2*-knockout mice suggests that TREM2 is a pivotal factor in this transition. Beyond AD, TREM2 has been implicated in multiple sclerosis [31] and neuropathic pain [32], and the R47H variant has also been reported as a risk factor for frontotemporal dementia and Parkinson’s disease [33].

Moreover, TREM2 is broadly expressed on the surface of cells of the monocyte–macrophage lineage and is implicated in diseases beyond the central nervous system. In obesity, lipid-associated macrophages (LAMs)—located in close proximity to hypertrophied adipocytes—exhibit TREM2-mediated regulation of the expression of genes related to phagocytosis, lipid catabolism, and energy metabolism [34]. In the absence of TREM2, LAM function is impaired, leading to adipocyte hypertrophy, hypercholesterolemia, and glucose intolerance [34]. Additionally, TREM2 has been identified as a marker of tumor-associated macrophages in various tumor types and influences tumor progression by modulating signaling pathways that govern cellular proliferation and metastasis [35]. Within the tumor microenvironment, TREM2 plays an immunosuppressive role, negatively regulating antitumor immune responses.

As outlined above, TREM2 expression plays a pivotal role in the involvement of microglia and macrophages in various disease processes. Several transcriptional regulators of the *TREM2* gene have been identified. The master microglial regulator PU.1 (SPI1) enhances the *TREM2* transcriptional activity, resulting in the promotion of microglial phagocytosis [36]. Moreover, Aβ clearance depends on the expression level of Spi1 [37], indicating that transcription factors themselves can modulate AD pathogenesis. This further implies that transcriptional regulators of *TREM2* may also serve as modulators of disease. The transcription factor YY1 has been recognized as the minimal promoter-binding factor of *TREM2* and has been shown to activate its expression in vitro [38]. Additionally, activation of the transcription factor Nrf2 promotes *TREM2* transcription and enhances the anti-inflammatory microglial phenotype characterized by Arginase-1 positivity, leading to behavioral improvements in a mouse model of depression [39]. Although many transcription factors are known to characterize microglial gene expression, only a limited number have been clearly shown to regulate TREM2. Identifying the regulatory factors governing *TREM2* transcription could lead to important insights into the mechanisms underlying microglial function and/or AD progression.

In the present study, we employed a reporter construct containing the upstream genomic region of the *TREM2* gene, including its promoter, to identify transcription factors—particularly those expressed in microglia—that promote *TREM2* expression. As a result, we identified ZEB2 as a novel regulatory factor of *TREM2*.

## 2. Materials and Methods

### 2.1. Plasmid Construction

KOD Plus Neo (TOYOBO, Tokyo, Japan) or KOD One (TOYOBO) was used for all the polymerase chain reactions (PCRs) performed for plasmid construction in this study. The primers used for plasmid construction are listed in Supplementary Table S1. The DNA sequences were verified by Sanger sequencing (Fasmac, Kanagawa, Japan).

#### 2.1.1. TREM2 Reporter Constructs

The T2-5k fragment was amplified from human genomic DNA (derived from THP-1 cells) by nested PCR using the primers nest-TREM2-pro-Fw and nest-TREM2-pro-Rv, followed by a second PCR with BamHI-TREM2-pro-Fw and SalI-TREM2-pro-Rv. The PCR product was digested with BamHI, blunt-ended using T4 DNA polymerase (TaKaRa, Shiga, Japan), and subsequently digested with SalI. The resulting fragment was inserted into the AseI (blunted)-SalI site of mCherry-N3.

To generate *T2-5k-u-mCherry*, the TREM2 5′UTR fragment was amplified from human genomic DNA (derived from THP-1 cells) using the primers NEB-TREM2-pro-PmaCI-Fw and NEB-TREM2-pro-HindIII-Rv. This fragment was inserted into the PmaCI-HindIII site of *T2-5k-mCherry* using NEBuilder (New England BioLabs, Ipswich, MA, USA).

The *T2-5k-u* fragment was amplified by PCR using the primers NheI-TREM2-pro-Fw and NEB-TREM2-pro-HindIII-Rv. After digestion with NheI and HindIII, the fragment was inserted into the NheI-HindIII site of pGL4.14 [luc2/Hygro] (Promega, Madison, WI, USA) to generate *T2-5k-u-Luc2*.

The *T2-2.5k-u* fragment was amplified by PCR using the primers AseI-TREM2-pro-2500-Fw and HindIII-TREM2-5′UTR-Rv, digested with AseI and HindIII, and blunt-ended only at the AseI site. This fragment was inserted into the EcoRV-HindIII site of pGL4.14.

To construct *T2-1k-u-Luc2*, the intermediate plasmid *T2-1k-u-mCherry* was generated by amplifying the fragment with AseI-TREM2-pro-1000-Fw and HindIII-TREM2-5′UTR-Rv, followed by digestion with AseI and HindIII. The fragment was inserted into the AseI-HindIII site of mCherry-N3. *T2-1k-u-Luc2* was then prepared by excising the *T2-1k-u-mCherry* fragment with AseI and HindIII, blunt-ending only the AseI site, and subcloning into the EcoRV-HindIII site of pGL4.14.

Substitutions in *T2-5k-u-mut-Luc2* were introduced by PCR using the following primer pairs: NEB-TREM2-pro-site1-Fw/Rv, NEB-TREM2-pro-site2-Fw/Rv, NEB-TREM2-pro-site3-Fw/Rv, NEB-TREM2-pro-site4-Fw/Rv, and NEB-TREM2-pro-site5-Fw/Rv.

#### 2.1.2. Enhanced Green Fluorescence Protein (EGFP)-Fused Transcription Factors

Fragments of each transcription factor were obtained by PCR using cDNA derived from THP-1 cells, human frontal cortex (Ambion, Austin, TX, USA), or the Lambda Zap II human adult brain cDNA library (Stratagene, Santa Clara, CA, USA). These fragments were inserted into pEGFP-C1 (Clontech, San Jose, CA, USA). For SMAD3, constitutively active mutations were introduced by substituting the four C-terminal amino acids, SSVS, with DDVD [40]. A point mutation and deletion constructs of ZEB2 were generated by PCR using specific primer sets, with the full-length ZEB2 expression vector serving as the template.

#### 2.1.3. Doxycycline-Inducible EGFP-ZEB2

The EGFP-ZEB2 fragment was excised from the EGFP-ZEB2 vector by digestion with NheI and SmaI, and inserted into the NheI–BamHI site of the PB-tet-EGFP-Azu-Puro vector [41], in which the BamHI site was blunt-ended using T4 DNA polymerase.

### 2.2. Cell Culture

The HEK293 (RCB1637) and THP-1 (RCB1189) cell lines were obtained from the RIKEN BioResource Research Center (Kyoto, Japan). The HMC3 cell line (EP-CL-0620) was obtained from Elabscience (Houston, TX, USA). HEK293 cells were maintained in Dulbecco’s Modified Eagle Medium (DMEM; Invitrogen, Waltham, MA, USA) supplemented with 10% fetal bovine serum (FBS; Nichirei Biosciences, Tokyo, Japan) and 1% penicillin–streptomycin (Wako, Osaka, Japan) at 37 °C in a humidified atmosphere containing 5% CO_2_. THP-1 cells were cultured in RPMI 1640 Medium with GlutaMAX supplement (Thermo Fisher Scientific, Waltham, MA, USA), supplemented with 10% FBS and 1% penicillin–streptomycin, under the same conditions. HMC3 cells were cultured in Eagle’s Minimum Essential Medium (EMEM; Wako, Tokoy, Japan), supplemented with 10% FBS and 1% penicillin–streptomycin, at 37 °C in 5% CO_2_. Inducible EGFP-ZEB2 cells were generated from THP-1 cells according to the protocol described in our previous study [41]. Previously established inducible EGFP cells were used as a negative control [41]. After 48 h of treatment with 1 μg/mL doxycycline (Sigma, St. Louis, MO, USA), cells were harvested and subjected to subsequent experiments.

### 2.3. Cellular Fluorescence Analysis Using IN Cell Analyzer 2000

THP-1 cells were seeded into 24-well plates on the day of transfection. A total of 0.25 μg of plasmid DNA was transfected using GeneXPlus (ATCC, Manassas, VA, USA). The following day, the culture medium was replaced. THP-1 cells were resuspended in medium containing 100 ng/mL phorbol 12-myristate 13-acetate (AdipoGen, San Diego, CA, USA) and redistributed from 1 well of the 24-well plate into 5 wells of 96-well plates. HMC3 cells were seeded into 96-well plates on the day of transfection. Cells were fixed with 4% paraformaldehyde (Wako) and stained with Hoechst 33342 (DOJINDO, Kumamoto, Japan). Fluorescence images of mCherry and Hoechst were acquired using the IN Cell Analyzer 2000 (GE Healthcare, Chicago, IL, USA). For each well, nine predefined fields were captured and analyzed.

### 2.4. Dual-Luciferase Reporter Assay

To perform the dual-luciferase assay, HEK293 cells were seeded in a 48-well plate one day prior to transfection. The cells in each well were co-transfected with 0.025 μg of a Photinus pyralis luciferase-fused TREM2 reporter plasmid, 0.1 μg of a cDNA construct, and 0.00025 μg of the Renilla reniformis luciferase plasmid (pGL4.75[hRluc/CMV], Promega) using Lipofectamine 3000 (Thermo Fisher Scientific). The pGL4.75[hRluc/CMV] plasmid served as an internal control. Forty-eight hours post-transfection, the cells were washed with PBS and lysed in 65 μL of Passive Lysis Buffer (Promega). The luciferase activity was measured using 4 μL of lysate, 20 μL of luciferase assay substrate, and 20 μL of Stop & Glo substrate. The luminescence was detected using a BioTek Synergy H1 microplate reader (Agilent, Santa Clara, CA, USA).

### 2.5. Quantitative Polymerase Chain Reaction (qPCR)

HMC3 cells were seeded into 12-well plates one day prior to transfection. siRNA was transfected using Lipofectamine RNAiMAX (Thermo Fisher Scientific) at a final concentration of 30 nM. Three days after transfection, total RNA was extracted and purified using either NucleoSpin RNA (Macherey-Nagel, Düren, Germany) or FastGene RNA Basic (Nippon Genetics, Tokyo, Japan), including DNase I treatment. Equal amounts of RNA were used for reverse transcription (RT) with ReverTra Ace (TOYOBO), using both random primers and oligo(dT) primers. The resulting cDNA was amplified using KAPA SYBR Fast qPCR Master Mix (Nippon Genetics), and the signals were detected with a LightCycler 96 system (Roche, Basel, Switzerland). siRNA targeting luciferase (Bioneer, Daejeon, Republic of Korea) was used as a negative control. The siRNA sequences targeting ZEB2 and the primers used for RT-qPCR are listed in Supplementary Tables S2 and S3, respectively.

### 2.6. Isolation of Membrane Fraction

The membrane-bound protein fraction was isolated according to a previously published protocol [42]. Briefly, cells were suspended in PBS and subjected to three freeze–thaw cycles (−80 °C for 1 h followed by thawing). The samples were then centrifuged at 16,000× *g* for 10 min, and the pellets were resuspended in buffer (20 mM Tris-HCl, 150 mM NaCl, 1 mM EDTA, 1% Triton X-100, protease inhibitor, pH 7.5). After 5 min incubation on ice, the samples were centrifuged again at 16,000× *g* for 10 min, and the supernatant was collected as the membrane-bound protein fraction. HMC3 cells were seeded into 6-well plates for siRNA transfection. Transfected cells from three wells were pooled and collected as a single sample for the preparation of the membrane-bound protein fraction. The protein concentration was determined using the BCA Protein Assay Kit (Thermo Fisher Scientific). APP was used as a loading control.

### 2.7. SDS-PAGE and Western Blotting

Total cell lysates were prepared by suspending cells in 2% SDS/PBS, followed by sonication. The protein concentration was determined using the BCA Protein Assay Kit (Thermo Fisher Scientific), and equal amounts of protein were mixed with SDS sample buffer and boiled. Proteins were separated on 10% polyacrylamide gels and transferred onto nitrocellulose membranes. Membranes were probed with appropriate primary antibodies and HRP-conjugated secondary antibodies. Blot images were captured using the Luminograph III system (ATTO, Tokyo, Japan), and the band intensities were quantified using ImageJ software (version 1.54, NIH, Bethesda, MD, USA). The following primary and secondary antibodies were used: Rabbit anti-ZEB2 (Cell Signaling Technology, #97885, Danvers, MA, USA), Rabbit anti-Amyloid Precursor Protein (abcam, ab32136, Cambridge, UK), Rabbit anti-TREM2 (Cell Signaling Technology, #91068), Mouse anti-GFP (proteintech, 66002-1-Ig, Rosemont, IL, USA), Goat anti-HSP60 (Everest Biotech, EB12834, Oxfordshire, UK), HRP-conjugated anti-Rabbit IgG (Jackson ImmunoResearch, 111-035-144, West Grove, PA, USA), HRP-conjugated anti-Mouse IgG (Jackson ImmunoResearch, 115-035-003), and HRP-conjugated anti-Rabbit IgG (Jackson ImmunoResearch, 705-035-003). Uncropped blot images are shown in Supplementary Figure S8.

### 2.8. Chromatin Immunoprecipitation (ChIP)

Approximately 6 × 10^6^ inducible EGFP or EGFP-ZEB2 cells were fixed with 1% paraformaldehyde to crosslink DNA–protein complexes. The crosslinking reaction was quenched by adding 0.25 M glycine. The fixed cells were resuspended in RIPA buffer containing 0.005% Triton X-100. Genomic DNA was sheared using a Bioruptor II (Sonicbio, Kanagawa, Japan) set to “high” power, applying six cycles of 30 s and 1 min off, while keeping the samples chilled in ice water throughout the sonication process. After sonication, the lysates were centrifuged at 16,000× *g* for 10 min at 4 °C. The supernatant was collected, and the protein concentration was determined using the BCA Protein Assay Kit (Thermo Fisher Scientific). A portion of the supernatant was reserved as an input sample, and the remaining supernatant was incubated overnight at 4 °C with anti-GFP antibody-conjugated magnetic beads (MBL, Tokyo, Japan) pre-equilibrated with RIPA buffer, under rotation. The magnetic beads were washed three times with RIPA buffer. To reverse crosslinking, ChIP elution buffer (10 mM Tris-HCl, pH 8.0; 300 mM NaCl; 5 mM EDTA, pH 8.0; 10% SDS) was added to the washed beads, followed by incubation at 65 °C for 4 h. The eluate was treated with RNase Cocktail (Ambion) at 37 °C for 30 min to degrade RNA. Proteinase K (Kanto Chemical, Tokyo, Japan) was then added, and the mixture was incubated at 60 °C for 1 h to digest proteins. DNA was subsequently purified using the NucleoSpin Gel and PCR Clean-up Kit (TaKaRa) according to the manufacturer’s instructions, and the recovered DNA was analyzed by qPCR. The primers used for the qPCR are listed in Supplementary Table S3.

### 2.9. Statistical Analysis

Statistical analyses were performed using R software (version 3.6.1). For comparisons between two groups, Welch’s two-tailed t-test was applied using Microsoft Excel (2016, version 2507). For comparisons among more than two groups, one-way ANOVA followed by a multiple comparison test (Tukey’s or Dunnett’s) was performed. In certain analysis, the data were analyzed using a non-parametric multiple comparison test (Steel–Dwass), due to the non-equal distribution of the data. *n* denotes the number of biological replicates. Error bars indicate standard deviations (SDs). The sample sizes and statistical tests are described in the figure legends. Each dot on the graph represents an individual data point.

## 3. Results

### 3.1. Construction and Evaluation of TREM2 Reporter Genes

**Figure 1:**
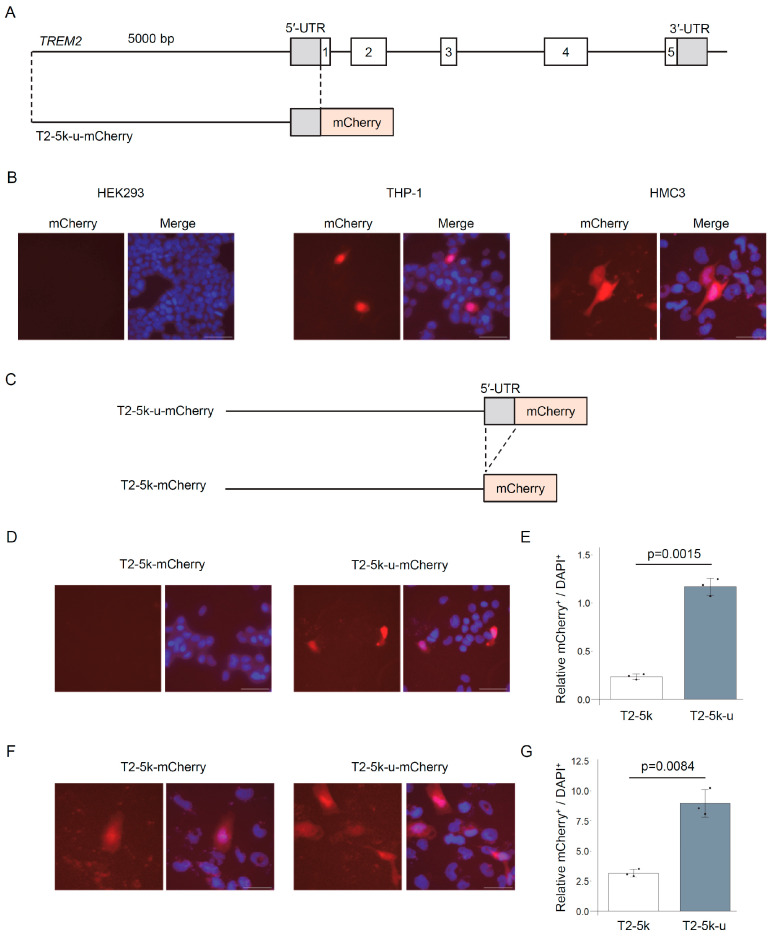
The 5 kb upstream region and 5′-UTR of *TREM2* recapitulate its cell type-specific expression. (**A**) Schematic representation of the reporter construct (*T2-5k-u-mCherry*). The CMV promoter in the mCherry-N3 vector was replaced with a fragment containing the 5 kb sequence upstream of the *TREM2* transcription start site and its 5′-UTR. (**B**) *T2-5k-u-mCherry* was transfected into HEK293, THP-1, and HMC3 cells. Representative fluorescence images of mCherry expression are shown. Nuclei were counterstained with Hoechst33342. Scale bars, 50 μm. (**C**) Schematic diagram of the *T2-5k-mCherry* construct lacking the *TREM2* 5′-UTR. (**D**) Fluorescent images of THP-1 cells transfected with *T2-5k-u-mCherry* or *T2-5k-mCherry*. Scale bars, 50 μm. (**E**) Quantification of mCherry-positive cells relative to Hoechst-positive cells using the IN Cell Analyzer. Error bars represent SDs; *n* = 3; Welch’s *t*-test. (**F**) Fluorescence images of HMC3 cells transfected with *T2-5k-u-mCherry* or *T2-5k-mCherry*. Scale bars, 50 μm. (**G**) Quantification of mCherry-positive cells relative to Hoechst-positive cells analyzed as in (**E**). Error bars represent SDs; *n* = 3; Welch’s *t*-test.

We fused the 5′ untranslated region (5′-UTR) of the human *TREM2* gene, along with its upstream 5 kb promoter region, to the upstream sequence of the red fluorescent protein mCherry (*T2-5k-u-mCherry*, Figure 1A). TREM2 is not expressed in HEK293 cells but is expressed in human monocyte-like THP-1 cells and human microglia-derived HMC3 cells. Upon the transfection of this construct into the cell lines, red fluorescence was observed in THP-1 and HMC3 cells, but not in HEK293 cells (Figure 1B), indicating that the 5 kb upstream region recapitulates the cell-type-specific expression of TREM2. Next, we compared the presence or absence of the 5′-UTR in THP-1 and HMC3 cells, both of which express endogenous TREM2. Deleting the 5′-UTR from the construct (*T2-5k-mCherry*, Figure 1C) markedly reduced mCherry expression, suggesting that the 5′-UTR is essential for the efficient expression of *TREM2* (Figure 1D–G). Therefore, in the following experiments, we used the upstream region of *TREM2*, including the 5′-UTR.

### 3.2. Exploration of Transcription Factors That Activate the TREM2 Reporter Gene

**Figure 2:**
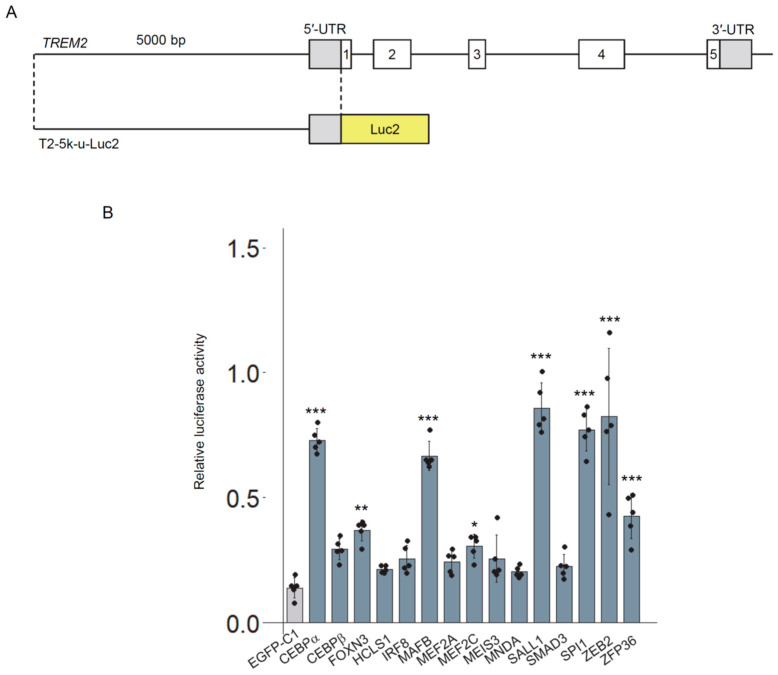
Evaluation of candidate transcription factors driving *TREM2* promoter activity using a luciferase assay. (**A**) Schematic representation of the reporter construct (*T2-5k-u-Luc2*) designed to assess *TREM2* promoter activity. (**B**) *T2-5k-u-Luc2* and transcription factor expression vectors were co-transfected into HEK293 cells, and luciferase activity was measured in cell lysates. Error bars indicate standard deviations (SDs); *n* = 5; statistical analysis by Dunnett’s test. *** *p* < 0.001, ** *p* < 0.005, * *p* < 0.05.

To enable a more quantitative assessment, we constructed a reporter plasmid (*T2-5k-u-Luc2*) by fusing the 5 kb upstream region of *TREM2* to the firefly luciferase gene (Figure 2A). Using this reporter, we evaluated the transcriptional activation potential of 15 transcription factors suggested to be expressed in microglia (Supplementary Table S4) [43]. Each transcription factor was expressed as an EGFP-fusion protein. The co-transfection of *T2-5k-u-Luc2* and each transcription factor into HEK293 cells revealed that several factors—CEBPa, FOXN3, MAFB, MEF2C, SALL1, SPI1 (which encodes PU.1), ZEB2, and ZFP36—significantly increased luciferase activity compared to the EGFP negative control (Figure 2B). Among the transcription factors that exhibited the greatest changes, CEBPα, MAFB, SALL1, and SPI1 have been reported in the context of microglia or TREM2 [36,44,45,46]. In addition, SALL1, which exhibited the highest transcriptional activation, was found to be undetectable in HMC3 cells. In contrast, ZEB2 has not been thoroughly investigated in the context of microglia and TREM2, highlighting the importance of focusing on this transcription factor. Moreover, a single-cell RNA-seq database [47] indicates that a larger proportion of TREM2-positive microglia co-express ZEB2 compared to other candidates and the known regulators SPI1 and YY1 (Supplementary Figure S1). Therefore, we focused on ZEB2 in subsequent analyses.

### 3.3. ZEB2-Mediated Regulation of TREM2 Expression

**Figure 3:**
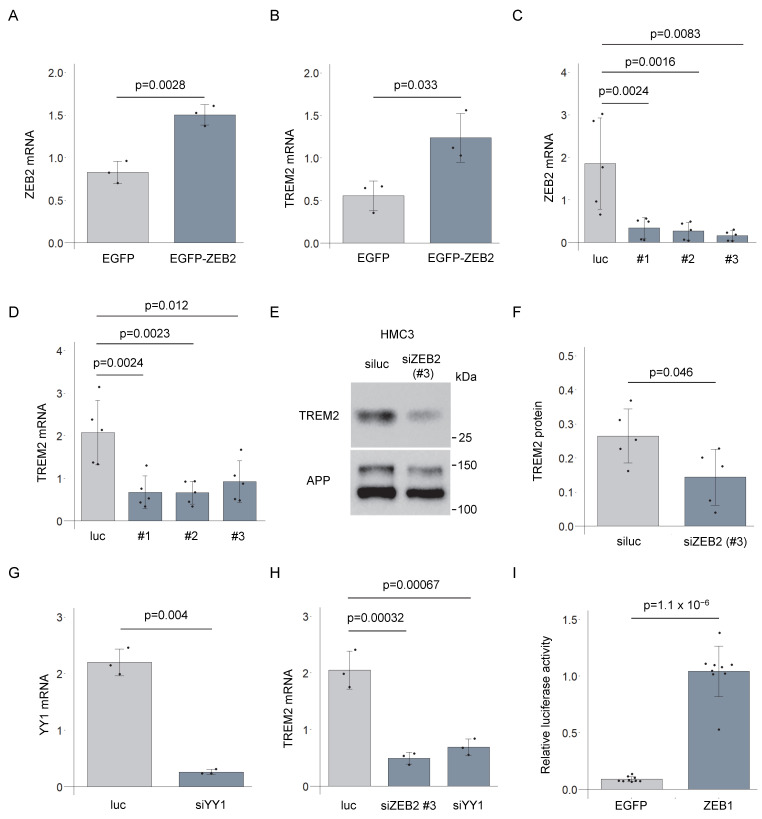
ZEB2 overexpression or knockdown modulates *TREM2* mRNA and protein expression. (**A**) RT-qPCR analysis of *ZEB2* mRNA in doxycycline-treated inducible EGFP and EGFP-ZEB2 cells. Expression was normalized to *ACTB*. Error bars represent SDs; *n* = 3; Welch’s *t*-test. (**B**) *TREM2* mRNA levels normalized to *ACTB*, quantified from the same samples as in (**A**). Error bars indicate SDs; *n* = 3; Welch’s *t*-test. (**C**) *ZEB2* siRNAs were transfected into HMC3 cells. *ZEB2* mRNA levels were measured by RT-qPCR and normalized to *B2M*. Error bars indicate SDs; *n* = 5; Tukey’s test. (**D**) *TREM2* mRNA levels quantified as in (**C**). Error bars indicate SDs; *n* = 5; Tukey’s test. (**E**) Membrane-bound protein fractions from HMC3 cells were analyzed for TREM2 protein levels following *ZEB2* knockdown. APP served as a loading control. (**F**) Quantification of (**E**). Error bars indicate SDs; *n* = 5; Welch’s *t*-test. (**G**) Relative *YY1* mRNA expression in HMC3 cells transfected with *YY1* siRNA. *YY1* mRNA levels were normalized to B2M. Error bars indicate SDs; *n* = 3; Welch’s test. (**H**) *TREM2* mRNA levels in HMC3 cells transfected with siZEB2 or siYY1. Error bars indicate SDs; *n* = 5; Tukey’s t-test. (**I**) Relative luciferase activity induced by EGFP-ZEB1. Error bars indicate SDs; *n* = 3; Welch’s test.

We used drug-inducible THP-1 cell lines capable of expressing either EGFP-ZEB2 or EGFP alone in a doxycycline (Dox)-dependent manner (Supplementary Figure S2). In the EGFP-ZEB2 cell line, Dox treatment led to an increase in *ZEB2* mRNA levels, accompanied by a concomitant upregulation of *TREM2* mRNA, relative to the EGFP cell line (Figure 3A,B). Although we attempted *ZEB2* knockdown in THP-1 cells, we were unable to confirm the effective suppression of *ZEB2* expression. As an alternative approach, we examined the endogenous expression of TREM2 in HMC3 cells. The knockdown of endogenous *ZEB2* using three distinct siRNAs resulted in a significant reduction in both *ZEB2* and *TREM2* mRNA levels across all siRNA treatments (Figure 3C,D). The expression of endogenous ZEB2 protein was inhibited by these siRNAs (Supplementary Figure S3). Furthermore, treatment with siZEB2 led to a decrease in TREM2 protein levels in HMC3 cells (Figure 3E,F). These findings suggest that ZEB2 functions as a transcriptional activator of TREM2 expression. YY1 was recently identified as a transcriptional regulator of *TREM2* [38]. We prepared siRNAs targeting *YY1* and examined changes in *TREM2* mRNA expression in HMC3 cells (Figure 3G). Both YY1 and ZEB2 knockdown reduced *TREM2* mRNA levels to a similar extent (Figure 3H).

We also examined the effect of ZEB1, a paralog of ZEB2. According to the Human Protein Atlas (https://www.proteinatlas.org/, accessed on 18 September 2025), ZEB1 is expressed in oligodendrocytes, microglia, and neurons. In the *TREM2* reporter assay, ZEB1 similarly enhanced transcriptional activity, comparable to the effect observed with ZEB2 (Figure 3I). We also compared the effects of EGFP-fused ZEB1 and ZEB2 on *TREM2* transcriptional activity and observed moderately higher luciferase activity with ZEB1 (Supplementary Figure S4A). This effect is likely attributable to the higher expression of ZEB1 than ZEB2 in HEK293 cells (Supplementary Figure S4B).

### 3.4. Deletion Analysis of ZEB2 Protein

**Figure 4:**
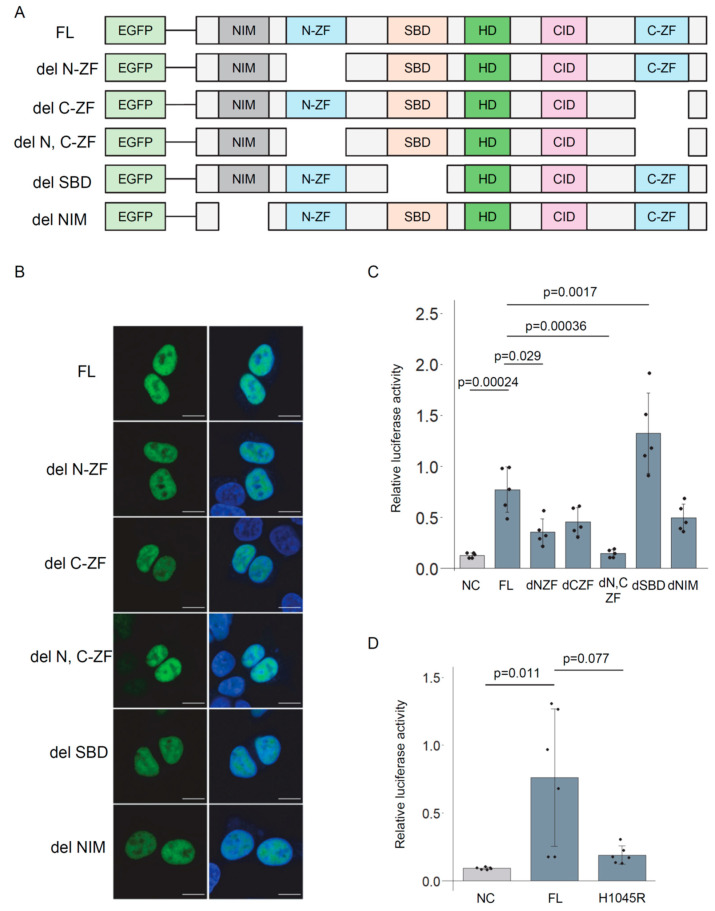
The zinc finger domains of ZEB2 contribute to the transcriptional regulation of *TREM2*. (**A**) Schematic representation of ZEB2 deletion mutants, each fused to the C-terminus of EGFP. N-ZF and C-ZF denote the N- and C-terminal zinc finger domains, respectively; NIM, NuRD-interacting motif; SBD, Smad-binding domain. (**B**) Intracellular localization of EGFP-fused ZEB2 mutants in HEK293 cells. Merged images show EGFP and DAPI overlays. Scale bars, 10 μm. (**C**) Luciferase assay of *T2-5k-u-Luc2* promoter activity following expression of ZEB2 mutants in HEK293 cells. EGFP was used as a negative control (NC). Error bars indicate SDs; *n* = 5; Tukey’s test. (**D**) Luciferase assay of *T2-5k-u-Luc2* following expression of the ZEB2 H1045R mutant, which resides within the C-ZF domain. Error bars indicate SDs; *n* = 6; Steel-Dwass test.

ZEB2 is a multi-domain protein [48]. To assess the contribution of individual domains to transcriptional activity, we compared constructs lacking specific domains, including the N-terminal and C-terminal zinc finger domains (N-ZF and C-ZF), the SMAD-binding domain (SBD), and the NuRD-interacting motif (NIM). A series of ZEB2 deletion mutants was generated (Figure 4A, Supplementary Figure S4B), and their transcriptional activity was evaluated using a reporter assay. All of these mutants exhibited nuclear localization (Figure 4B). Deletion of the N-ZF resulted in a decrease in luciferase activity, and deletion of both zinc fingers led to a further reduction. (Figure 4C). Removal of the SBD led to increased activity. *ZEB2* is a causative gene for Mowat–Wilson syndrome [49,50]. The H1045R mutation in *ZEB2* is a pathogenic variant associated with the disease and is located within the C-ZF domain [51], where it is predicted to disrupt zinc finger structure [52]. The H1045R mutant tended to exhibit lower transcriptional activity compared to the wild-type (WT) protein (Figure 4D). These findings suggest that both of the ZF domains of ZEB2 are essential for the transcriptional activation of the *TREM2* gene.

### 3.5. TREM2 Upstream Region Is Bound by ZEB2

**Figure 5:**
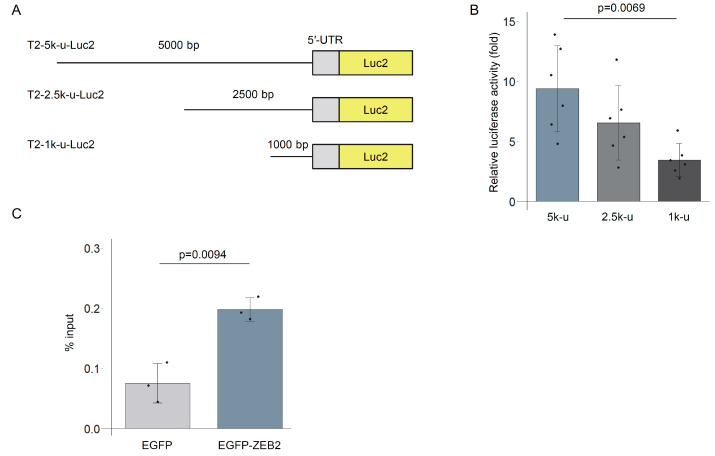
The 5 kb upstream sequence of *TREM2* mediates ZEB2-driven transcriptional activity. (**A**) Schematic representation of truncated reporter constructs used to evaluate ZEB2 responsiveness within the *TREM2* upstream sequence. (**B**) Luciferase assay of promoter activities of the truncated constructs following ZEB2 expression. Bar graph shows the fold increases in luciferase activity upon EGFP-ZEB2 introduction compared with EGFP alone for each reporter construct. Error bars indicate SDs; *n* = 6, Tukey’s test. (**C**) ChIP-qPCR analysis of ZEB2 association with the *TREM2* upstream region. EGFP or EGFP-ZEB2 was immunoprecipitated from inducible cell lines using an anti-GFP antibody. Bound DNA was quantified by qPCR using primers targeting the *TREM2* upstream region. Error bars indicate SDs; *n* = 3; Welch’s *t*-test.

We investigated the binding of ZEB2 to the upstream region of *TREM2*. To this end, we generated a series of reporter constructs containing truncated versions of the upstream region (Figure 5A). Progressive deletion from 5 kb to 1 kb upstream of *TREM2* significantly attenuated ZEB2-mediated transcriptional activation (Figure 5B), although residual responsiveness to ZEB2 remained. In contrast, reporter constructs lacking any *TREM2* sequence did not exhibit transcriptional activation by ZEB2 (Supplementary Figure S5), indicating that the regulation is dependent on the *TREM2* upstream region. ZEB2 is known to recognize the CACCTG motif [53], and multiple similar motifs were identified within the upstream sequences (Supplementary Figure S6A).

To assess the physical interaction between ZEB2 and the *TREM2* upstream region, we performed chromatin immunoprecipitation (ChIP). DNA was recovered from THP-1 cells expressing either EGFP or EGFP-ZEB2 following immunoprecipitation with an anti-GFP antibody, and the upstream region of *TREM2* was quantified by qPCR. A significant enrichment of the *TREM2* upstream sequence was observed in EGFP-ZEB2–expressing cells (Figure 5C). Within the 5 kb upstream region, multiple CACCTG or CACCTG-like motifs were identified. The mutation of all five candidate motifs on the sense strand in the reporter construct markedly reduced ZEB2-induced transcriptional activation (Supplementary Figure S6B,C). Consistently, we observed multiple peaks in the upstream region of *TREM2* in a ZEB2 ChIP-seq profile of K562 leukemia-derived cells [54] (Supplementary Figure S7). Collectively, these findings suggest that ZEB2 binds to the upstream region of *TREM2* and promotes its transcriptional activity.

## 4. Discussion

In this study, we found that a construct containing the 5 kb upstream region and 5′-UTR of *TREM2* recapitulated its endogenous expression pattern. We also found that the 5′-UTR contributes to the gene’s transcriptional activation. Our previous work demonstrated that the 5′-UTR sequence plays a role in the species-specific regulation of *TREM2* translation [55]. Further investigation is needed to elucidate the precise mechanisms by which the 5′-UTR regulates both transcription and translation.

We found that overexpression of the transcription factor ZEB2 enhanced *TREM2* promoter activity (Figure 2), while the knockdown of ZEB2 reduced the levels of *TREM2*′s mRNA and protein (Figure 3). ZEB2 has not previously been implicated in *TREM2* regulation or in neurodegenerative diseases such as Alzheimer’s disease. In mice, *Zeb2* is one of the core genes expressed in pre-macrophages [56]. It is broadly expressed among tissue-resident macrophages and is required for maintaining their tissue-specific identities, including that of microglia [57]. A ZEB2 ortholog promotes the expansion of microglia in the brain development of zebrafish [58], but its role in human microglia remains unclear. It is best known for its role in epithelial–mesenchymal transition (EMT) [59,60], a process critical for embryonic development, wound healing, and cancer progression [61]. Conditional knockout of *Zeb2* in astrocytes attenuates astrogliosis in mice [62]. Haploinsufficiency of *ZEB2* causes Mowat–Wilson syndrome through abnormalities in neural crest cell differentiation, brain development, and the enteric nervous system, while the involvement of microglia or macrophages remains unclear [63]. Although ZEB2 is generally considered a transcriptional repressor [64], there are reports of ZEB2-mediated transcriptional activation [65,66], which are consistent with our current findings. The activation of *TREM2* transcription by ZEB2 might also be regulated by an unidentified cofactor. As *Zeb2*-deficient mice exhibit embryonic lethality [67], microglia-specific *Zeb2*-knockout mice are expected to be a valuable tool.

Our results also revealed that ZEB1 upregulates *TREM2* promoter activity. ZEB1 is structurally similar to ZEB2 and binds to E-box motifs, like ZEB2 [68], implicating an overlapping role in *TREM2* activation. In an acute ischemic stroke model, increased expression of ZEB1 reduces microglial reactivity and ameliorates brain damage [69]. Thus, ZEB1 is an interesting candidate that regulates microglial phenotypes; however, its involvement in *TREM2* regulation needs further characterization. Similarly, we observed increased luciferase activity upon the overexpression of CEBPA, MAFB, and SALL1, together with the known regulator SPI1. Although these factors are also candidates for regulating TREM2, experimental validation is needed to confirm their roles.

Analysis of single-cell RNA-seq data from human microglia [47] highlighted similar expression patterns of *SPI1* and *TREM2* among subpopulations (Supplementary Figure S1A). Notably, *ZEB2* expression was also correlated with that of *TREM2* in certain microglial subpopulations (clusters 2, 6, and 8, Supplementary Figure S1A), suggesting a role of ZEB2 in the condition-specific regulation of *TREM2*. The proportion of cells co-expressing *ZEB2* and *TREM2* was higher than that of cells co-expressing *SPI1*, *YY1*, or *ZEB1*, indicating that ZEB2 may promote *TREM2* expression in particular microglial subsets (Supplementary Figure S1B). In contrast, *YY1* and *ZEB1* exhibited expression patterns distinct from that of *TREM2*. There may be a regulatory hierarchy among these transcription factors.

Our results suggest that the zinc finger domains are critical for the ZEB2-mediated activation of *TREM2* transcription. In contrast, deletion of the Smad-binding domain resulted in a significant increase in *TREM2* promoter activity. Previous studies have shown that ZEB2 suppresses downstream gene expression through interactions with SMAD proteins [53]. Whether the ZEB2–SMAD interaction modulates *TREM2* expression remains an intriguing question for future investigation. These findings imply that multiple domains of ZEB2 contribute differently to *TREM2* transcriptional regulation, and that the functional balance between these domains is an important determinant of ZEB2-mediated regulation.

To further explore ZEB2-responsive elements within the upstream region of the *TREM2* gene, we employed a series of promoter deletion constructs (Figure 5). Progressive deletion of the upstream sequences led to a corresponding decrease in promoter activity, suggesting that multiple ZEB2-responsive elements are present and contribute cumulatively to transcriptional activation. Point mutations that altered predicted ZEB2-binding motifs in the upstream region of *TREM2* resulted in reduced promoter activity, although complete loss of ZEB2 responsiveness was not observed. One possible explanation is that the antisense strand still retained ZEB2-binding motifs in the mutant constructs, which may have contributed to residual transcriptional activation. Taken together, these findings suggest that the upstream region of *TREM2* contains multiple ZEB2-responsive elements that act in an additive manner to regulate transcription. Further studies are required to precisely map and characterize the ZEB2-binding motifs within this region.

One limitation of this study is that the experiments were conducted using immortalized cultured cell lines, and the findings have not been validated in microglia or in vivo. Another limitation is that the precise binding sites of ZEB2 in the upstream region of *TREM2* remain unidentified, and indirect binding cannot be ruled out. Additionally, the present study does not provide data that allow us to discuss the physiological and pathological significance of the ZEB2-mediated regulation of *TREM2* transcription. These issues will be addressed in our future studies.

## 5. Conclusions

In this study, our findings suggest ZEB2 as a novel transcriptional regulator of *TREM2*. The expression of *TREM2* not only characterizes key cell types involved in the pathogenesis of several diseases, but is also thought to influence their functional roles. Our findings are expected to contribute to the molecular understanding of *TREM2* regulation. Future studies will be required to elucidate how the ZEB2-mediated control of *TREM2* expression contributes to physiological and pathological processes.

## Data Availability

All data are available from the corresponding authors upon reasonable request.

## Supplementary Materials

The following supporting information can be downloaded at: https://www.mdpi.com/article/10.3390/genes16111329/s1, Table S1: Primers used for plasmid construction; Table S2: siRNA sequences used in this study; Table S3: Primers used for qPCR; Table S4: Expression levels of transcription factors used in this study in microglia; Figure S1: Co-expression of transcription factors with TREM2 in microglial subpopulations; Figure S2: Establishment of inducible EGFP-ZEB2 cell lines; Figure S3: All three siZEB2 suppress endogenous ZEB2 protein expression; Figure S4: ZEB family enhances TREM2 transcriptional activity; Figure S5: Luciferase activity was not upregulated by ZEB2 in the absence of the TREM2 upstream sequence; Figure S6: The TREM2 upstream region contains ZEB2-binding motifs; Figure S7: ZEB2 ChIP-seq suggests the binding of ZEB2 to the upstream region of TREM2; Figure S8: Original uncropped images.

## Abbreviations

The following abbreviations are used in this manuscript:

Aβ	amyloid-β
AD	Alzheimer’s disease
ChIP	chromatin immunoprecipitation
DAM	disease-associated microglia
EGFP	enhanced green fluorescence protein
FBS	fetal bovine serum
LAM	lipid-associated macrophage
NIM	NuRD-interacting motif
PCR	polymerase chain reaction
qPCR	quantitative polymerase chain reaction
RT	reverse transcription
SBD	SMAD-binding domain
siRNA	small interfering RNA
TREM2	Triggering receptor expressed on myeloid cells 2
TYROBP	TYRO protein tyrosine kinase-binding protein
UTR	untranslated region
ZF	zinc finger
