# Supplementary material for: Transcriptional Activation of the TREM2 Gene by ZEB2 in a Zinc Finger-Dependent Manner
Source: Genes (Basel). 2025 Nov 3;16(11):1329. doi: 10.3390/genes16111329 (PMC12652027; doi:10.3390/genes16111329)
Supplement: Supplementary file 1 [file genes-16-01329-s001.zip › genes-3848259-supplementary.pdf]

Supplementary Table S1

Primer	Sequence (5′ to 3′)
nest-TREM2-pro-Fw	GCAGGAGGATGGAAGGAAGATTTCAGGGAGAAATA
nest-TREM2-pro-Rv	CTGTGACAAAGAGTAAGATGAGCAGCCGGAGAGGC
BamHI-TREM2-pro-Fw	AAAAGGATCCCTTTTCCCTCATATGCAGGCTGGAT
SalI-TREM2-pro-Rv	AAAAGTCGACGGCGCTGCCCACAGGAACTGGGCTC
NEB-TREM2-pro-PmaCI-Fw	TTTTGTGCCTTTGCACACGTGGGTCCCTGTGCTTGGATC ACCATGGGAAC
NEB-TREM2-pro-HindIII-Rv	GTACCGGATTGCCAAGCTTGGCCGCCGAGGCCAGATCT TGATATCCTCGACGCCACCCTTCCCCAGCCAAGGGCA
NheI-TREM2-pro-Fw	AAAAGCTAGCCTTTTCCCTCATATGCAGGCTGGAT
AseI-TREM2-pro-2500-Fw	AAAAATTAATGCCAACGTGGTGAAACCCCGTCTCT
AseI-TREM2-pro-1000-Fw	AAAAATTAATACTCCATTTCCCACTCCTAGAATTG
HindIII-TREM2-5′UTR-Rv	AAAAAAGCTTGCCACCCTTCCCCAGCCAAGGGCAG
NEB-TREM2-pro-5000-Fw	GTACCTGAGCTCGCTAGCCTTTTCCCTCATATGCA
NEB-TREM2-pro-site1-Fw	CCAGGCTTAGCAAAAGGGTTCATCTCATTTCCTGA
NEB-TREM2-pro-site1-Rv	CCCTTTTGCTAAGCCTGGTGCAGACATCGCAGGCA
NEB-TREM2-pro-site2-Fw	GTCCTGACCAACAAAAAGTGCTTTCCTGGCTGCTC
NEB-TREM2-pro-site2-Rv	CTTTTTGTTGGTCAGGACCATGGCCAGTGCCAGCC
NEB-TREM2-pro-site3-Fw	CCAGGCAGATCAAAAGAGGTCACGAGTTCGAGACC
NEB-TREM2-pro-site3-Rv	CTCTTTTGATCTGCCTGGCTCAGACTCCCAAAGTG
NEB-TREM2-pro-site4-Fw	GAAGGAGATATCAAAATAAACCTATTAGAAAGGCT
NEB-TREM2-pro-site4-Rv	TATTTTGATATCTCCTTCAATTTTAACTTGTATTT
NEB-TREM2-pro-site5-Fw	GAAAGCACTTTAAAAGTAGGAACCCATATGGTGCT
NEB-TREM2-pro-site5-Rv	TACTTTTAAAGTGCTTTCCAGTAGGGCCTGACACA
nest-CEBPA-Fw	GGGCGACGCGGCCTGCCGGGTATAAAAGCT
nest-CEBPA-Rv	GATCCGGCGACCCCAAACCACTCCCTGGGT
BglII-CEBPA-Fw	AAAAAGATCTATGGAGTCGGCCGACTTCTACGAGG
SalI-CEBPA-Rv	AAAAGTCGACTCACGCGCAGTTGCCCATGGCCTTG
nest-CEBPB-Fw	AGCAGCAGCAGCGACGCAGCGGCGACAGCTCAG
nest-CEBPB-Rv	AGTGCCAAAGTTTTGCCGGCGCCGCCGGGG
BglII-CEBPBFw	AAAAAGATCTATGCAACGCCTGGTGGCCTGGGACC
SalI-CEBPB-Fw	AAAAGTCGACCTAGCAGTGGCCGGAGGAGGCGAGC
nest-FOXN3-Fw	CAGCCCCCCAATGCGGCCGCGAGAAGCAGCGG
nest-FOXN3-Rv	ATGCTGAAACCAAATGGTCGTAAGTTCAAAACAA
BglII-FOXN3-Fw	AAAAAAGATCTATGGGTCCAGTCATGCCTCCCAGT
SalI-FOXN3-Rv	AAAAAAGTCGACTTAATTTTTTGTGGTTTCCTTTT
nest-HCLS1-Fw	CGGGCGCTTAGAACAGAGGCTTGCACAGGTGGAG
nest-HCLS1-Rv	AGCCACTTTGGACATGGGAAATCACAGTTGCAGTA
BglII-HCLS1-Fw	AAAAAAGATCTATGTGGAAGTCTGTAGTGGGCCAT
SalI-HCLS1-Rv	AAAAAAGTCGACTCACTCCAGAAGCTTGACATAAT
nest-IRF8-Fw	GTGAGGTCATGGAGGCCAGCATTGCCTTCTC
nest-IRF8-Rv	CCAGACAGAGGGATCCACATTCTTTAATCAT
BglII-IRF8-Fw	AAAAAAGATCTATGTGTGACCGGAATGGTGGTCGG
SalI-IRF8-Rv	AAAAAAGTCGACTTAGACGGTGATCTGTTGGTTTT
nest-MAFB-Fw	CGCGCTCCGGCCGGCCGCAAAGTTTCCC
nest-MAFB-Rv	TTTGTATTTTTCTATATAATCGAGCAGGCA
BglII-MAFB-Fw	AAAAAAGATCTATGGCCGCGGAGCTGAGCATGGGG
SalI-MAFB-Rv	AAAAAAGTCGACTCACAGAAAGAACTCGGGAGAGG

Primer	Sequence (5′ to 3′)
nest-MEF2A-Fw	TAACAGAAGCTGTGTACGATGCATTAGGGT
nest-MEF2A-Rv	CACTTCCTTGGACTACTTGTTTCGTAAAGA
BclI-MEF2A-Fw	AAAATGATCAATGGGGCGGAAGAAAATACAAATCA
SalI-MEF2A-Rv	AAAAGTCGACTTAGGTCACCCACGCGTCCATCCTC
nest-MEF2C-Fw	AATACATAATTTCAGGGACGAGAGAGAGAAGA
nest-MEF2C-Rv	TTCCACACACGGCACATATAATGCATATCG
BamHI-MEF2C-Fw	AAAAAGGATCCATGGGGAGAAAAAAGATTCAGATT
XhoI-MEF2C-Rv	AAAAACTCGAGTCATGTTGCCCATCCTTCAGAAAG
nest-MEIS3-Fw	ACTATTGTGGAAGAAGCATCCATTAAGGCCAAGC
nest-MEIS3-Rv	CACAGTTATTAACAACTTAATTGTTTTAAGCGGAA
BamHI-MEIS3-Fw	AAAAAGGATCCATGGCCCGGAGGTATGATGAGCTG
SalI-MEIS3-Rv	AAAAAGTCGACCTATAGATAATGCCATTCTCCTT
nest-MNDA-Fw	GAAGCATCCATTAAGGCCAAGCTATAACATCAGA
nest-MNDA-Rv	CTTAATTGTTTTAAGCGGAAGTTGTTTGTTGCAT
BglII-MNDA-Fw	AAAAAAGATCTATGGTGAATGAATACAAGAAAATT
SalI-MNDA-Rv	AAAAAAGTCGACTCAATTAACATTCATTGGTCCTT
nest-SALL1-Fw	ATTCTGCCCCAGCTGATGTTTGAGCCAGCA
nest-SALL1-Rv	TAGGTCGCATTCTGAACAGGAATGAATGCTATGTC
BspEI-SALL1-Fw	AAAAATCCGGAATGTCGCGGAGGAAGCAAGCGAAG
SalI-SALL1-Rv	AAAAAAGTCGACTTAACTCGTGACGATCTCCTCTTGC
nest-SMAD3-Fw	CGGCAACTTCGCCGAGAGTTGAGGCGAAGTTTG
nest-SMAD3-Rv	CTCCTGCATGGCCATTTTCCCCAAGCCTGCCCTCC
BamHI-SMAD3-Fw	AAAAAGGATCCATGTCGTCCATCCTGCCTTTCACT
SalI-SMAD3-CA-Rv	AAAAAAGTCGACCTAATCCACATCGTCACAGCGGATGC TTGGGGAGCCCATC
BglII-SPI1-Fw	AAAAAGATCTATGTTACAGGCGTGCAAAATGGAAG
SalI-SPI1-Rv	AAAAGTCGACTCAGTGGGGCGGGTGGCGCCGCTCG
nest-ZEB1-Fw	AGACACAAGCGAGAGGATC
nest-ZEB1-Rv	TGAAAAGCAAGGATAATATTG
BamHI-ZEB1-Fw	AAAAGGATCCATGGCGGATGGCCCCAGGTGTAAGC
SalI-ZEB1-Rv	AAAAGTCGACTTAGGCTTCATTTGTCTTTTCTTCA
nest-ZEB2-Fw	GTCCATGCGAACTGCCATCTGATCCGCTCTTATCA
nest-ZEB2-Rv	CAGCAGTGTTTTCAAGCAGGTAACAATACTACTGG
BglII-ZEB2-Fw	AAAAAAGATCTATGAAGCAGCCGATCATGGCGGAT
SalI-ZEB2-Rv	AAAAAGTCGACTTACATGCCATCTTCCATATTGT
BglII-ZFP36-Fw	AAAAAGATCTATGGCCAACCGTTACACCATGGATC
XhoI-ZFP36-Rv	AAAACTCGAGTCACTCAGAAACAGAGATGCGATTG
ZEB2-del-NZF-Fw	CCAACTGATTGGTTTAATCTCTGTAAATGGCCGAA
ZEB2-del-NZF-Rv	AAACCAATCAGTTGGGCAAAAGCATCTGGAGTTCC
ZEB2-del-CZF-Fw	AGAGTGGCTCCTACTGCAAGCGGGAGGCGGAGGAG
ZEB2-del-CZF-Rv	AGTAGGAGCCACTCTCTGTCTTCTTGATCTTTTTG
ZEB2-delSBD-Fw	CAGAGTCCAATGGGTTATCACATGAAGGATCCATG
ZEB2-delSBD-Rv	TGTGATAACCCATTGGACTCTGAGCAGATGGATGA
ZEB2-delNIM-Fw	GTGCAAGAAAAACGTGGTGAACTATGACAA
ZEB2-delNIM-Rv	ACGTTTTTCTTGCACCGGGGGCCATCCGCC
ZEB2-H1045R-Fw	ATCGAGCGCTCAAGGCTTCACTCGGGCGAG
ZEB2-H1045R-Rv	CCTTGAGCGCTCGATAAGGTGGTGCTTGTG

Supplementary Table S1. Primers used for plasmid construction.

Supplementary Table S2

siRNA	Sequence of sense strand (5′ to 3′)
ZEB2 #1	GACUAAUUCCUGUGUUUAAdTdT
ZEB2 #2	CAACAACGAGAUUCUACAAdTdT
ZEB2 #3	GCACAUCAGCAGCAACAAAdTdT
YY1	CGACGACUACAUUGAACAAdTdT

Supplementary Table S2. siRNA sequences used in this study.

Supplementary Table S3

Primer	Sequence (5′ to 3′)
ACTN-Fw	GAGCGCCATGAACCAGATAGA
ACTN-Rv	AGTGAAGGTCTTCCTCTGCTG
B2M-Fw	GAGTGCTGTCTCCATGTTTGATGT
B2M-Rv	AAGTTGCCAGCCCTCCTAGAG
TREM2-Fw	TCTGAGAGCTTCGAGGATGC
TREM2-Rv	GGGGATTTCTCCTTCCAAGA
ZEB2-Fw	CTAACCCAAGGAGCAGGTAATC
ZEB2-Rv	GTGAATTCGCAGGTGTTCTTTC
ChIP-Fw	CAGCACTTTGGGAGTCTGAG
ChIP-Rv	ACCACGCCTGGCTAATTT
YY1-Fw	GGATAACTCGGCCATGAGAAA
YY1-Rv	GAAAGGGCTTCTCTCCAGTATG

Supplementary Table S3. Primers used for qPCR.

Supplementary Table S4

**Transcription factor**	**average TPM (ref R1)**	**Refs Supporting Expression or Function in Microglia**	**Luc activity**
CEBPA	138	R1	**0.7302**
CEBPB	199.5	R1	0.2942
FOXN3	36.6	R4	**0.3688**
HCLS1	243.8	R2	0.2142
IRF8	182.5	R1, R2	0.2548
MAFB	131.6	R1	**0.6668**
MEF2A	205.6	R1	0.2448
MEF2C	108.2	R1	**0.3058**
MEIS3	0.1	R3	0.2568
MNDA	91.8	R2	0.2036
SALL1	90.4	R1, R3	**0.8588**
SMAD3	23	R1	0.2262
SPI1 (PU.1)	257.1	R1, R2	**0.7718**
ZEB2	37.8	R1	**0.8244**
ZFP36	860.9	R2	**0.4262**

Supplementary Table S4. Expression levels of transcription factors used in this study in microglia. The average transcripts per million (TPM) values of transcription factors in ex vivo microglia were obtained from Gosselin et al. (ref R1). The listed references support the expression or function of each transcription factor in microglia. The right column presents luciferase activity values corresponding to Fig. 2B, with statistically significant increases highlighted in bold.

R1: Gosselin *et al*. An environment-dependent transcriptional network specifies human microglia identity. *Science* **2017**, *356*, eaal3222.
R2: Galatro *et al*. Transcriptomic analysis of purified human cortical microglia reveals age-associated changes. *Nature Neuroscience* **2017**, *20,* 1162-1171.
R3: Mass *et al*. Specification of tissue-resident macrophages during organogenesis. *Science* **2016**, *353*, aaf4238.
R4: Li et al. Single-cell analysis reveals transcriptomic reprogramming in aging primate entorhinal cortex and the relevance with Alzheimer's disease. *Aging Cell* **2022**, *21*, e13723.

Supplementary Figure S1

A

	Cell number	percent
both	5470	6.03
TREM2	25826	28.47
CEBPA	5421	5.98
both	6285	6.93
TREM2	25011	27.57
MAFB	5762	6.35
both	4600	5.07
TREM2	26696	29.43
SALL1	5625	6.20
both	14086	15.53
TREM2	17210	18.97
SPI1	12024	13.25

	Cell number	percent
both	6287	6.93
TREM2	25009	27.57
YY1	8545	9.42
both	7226	7.97
TREM2	24070	26.53
ZEB1	13872	15.29
both	19758	21.78
TREM2	11538	12.72
ZEB2	40072	44.17

B

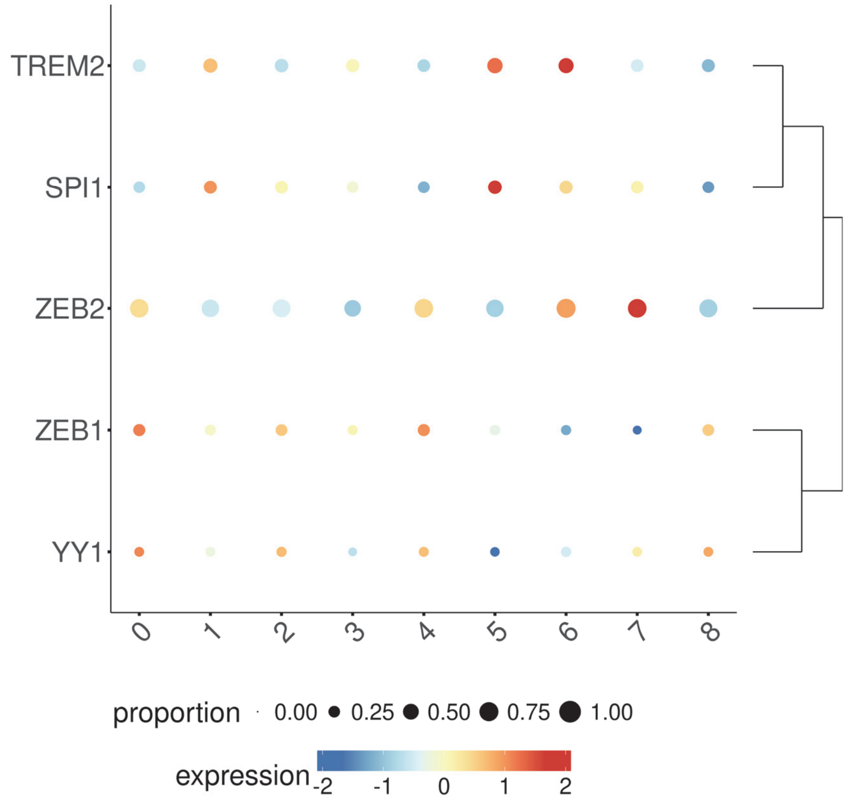

Supplementary Figure S1. **Co-expression of transcription factors with *TREM2* in microglial subpopulations.** Data were obtained from a publicly available meta-analysis of human microglial single-cell RNA-seq (Martins-Ferreira et al.; https://in7yqx-ricardo-ferreira.shinyapps.io/shinyapp/). **(A)** Higher proportion of microglia co-expressing *TREM2* and *ZEB2* compared with other transcription factors. **(B)** Correlation between the expression of each transcription factor and *TREM2* across individual microglial subpopulations. Clusters: 0, Homeos1 (Homeostasis); 1, Inflamm.DAM; 2, DIM (disease-inflammatory macrophages); 3, Ribo.DAM1; 4, Homeos2; 5, Ribo.DAM2; 6, Lipo.DAM; 7, MAC (macrophages); 8, Homeos3.

Supplementary Figure S2

A

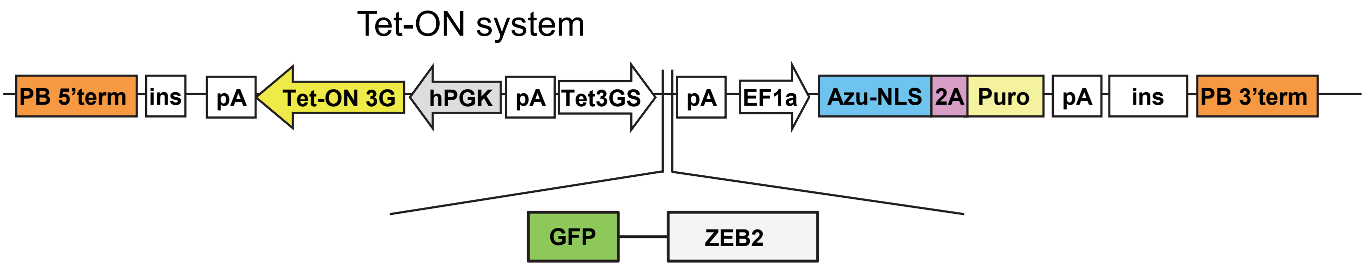

B

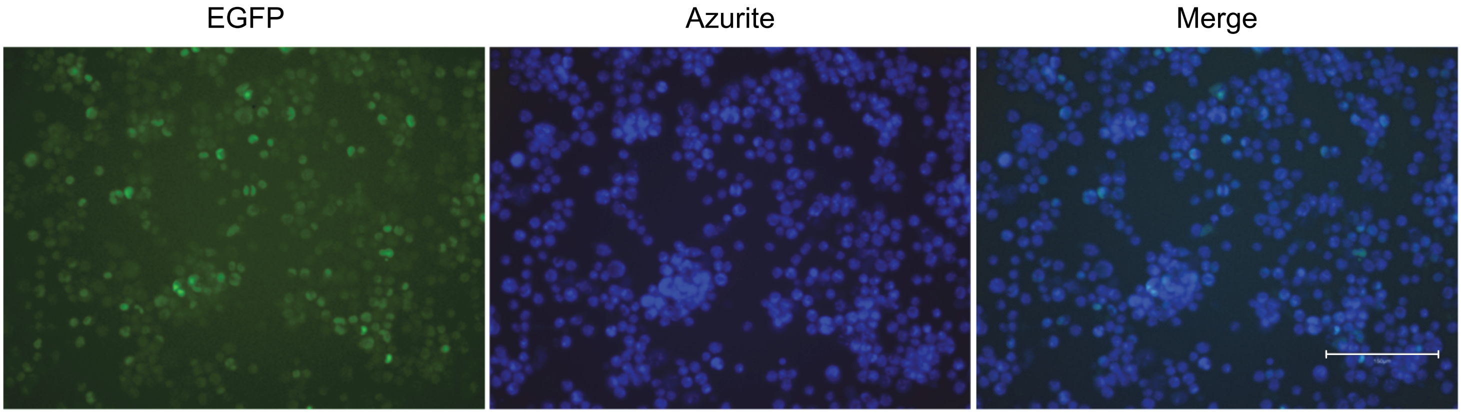

Supplementary Figure S2. Establishment of inducible EGFP-ZEB2 cell lines.

(**A**) Schematic representation of the inducible EGFP-ZEB2 vector.

(**B**) Inducible EGFP-ZEB2 THP-1 cells were established by puromycin selection and Azurite-derived blue fluorescence. Scale bar, 150 μm.

Supplementary Figure S3

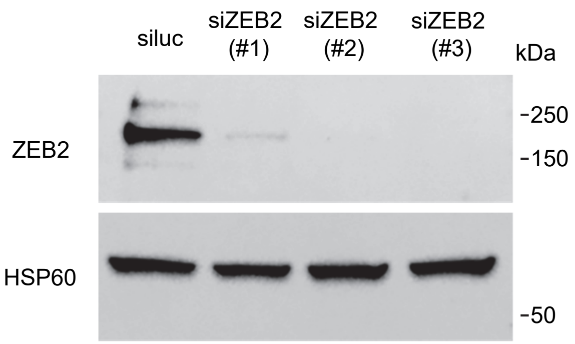

Supplementary Figure S3. All three siZEB2 suppress endogenous ZEB2 protein expression. All siZEB2 used in this study were transfected into HMC3 cells. Total cell lysates were subjected to SDS-PAGE and western blotting. HSP60 was used as a loading control.

Supplementary Figure S4

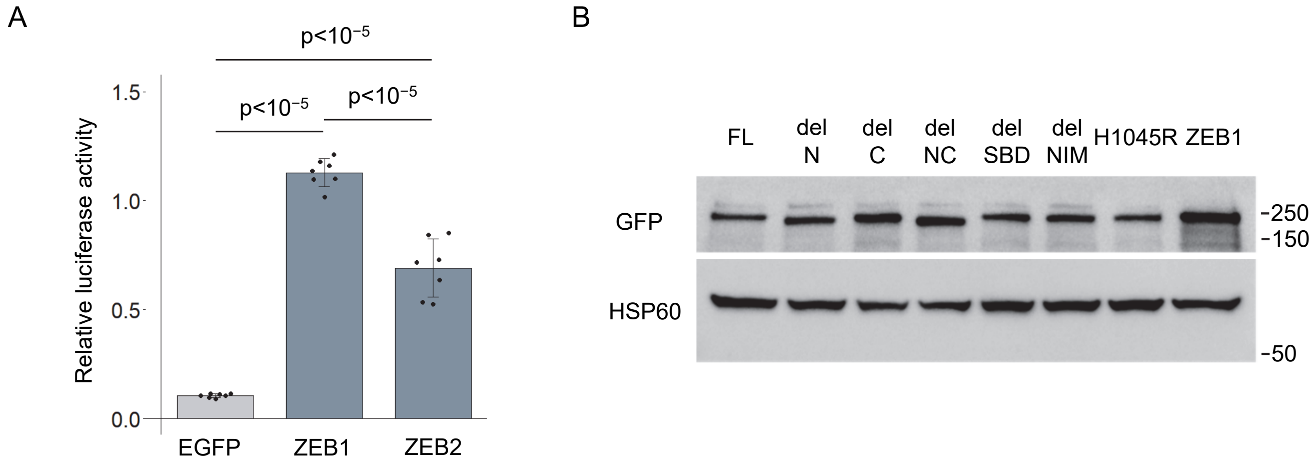

Supplementary Figure S4. ZEB family enhances *TREM2* transcriptional activity.
(A) EGFP-ZEB1 and EGFP-ZEB2 were co-transfected with T2-5k-u-Luc2 into HEK293 cells. Error bars indicate SD; $n = 7$; Tukey's test.

(B) EGFP-fused ZEB2 mutants or EGFP-ZEB1 were transfected into HEK293 cells. Total cell lysates were analyzed by SDS-PAGE and western blotting. HSP60 was used as a loading control.

Supplementary Figure S5

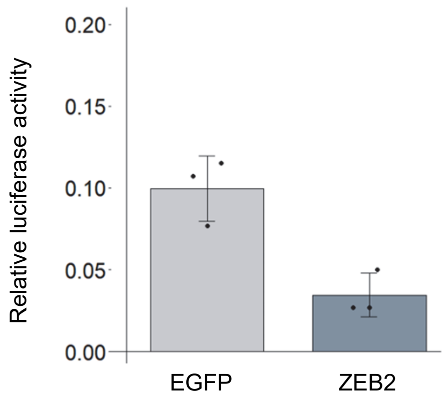

Supplementary Figure S5. Luciferase activity was not upregulated by ZEB2 in the absence of the *TREM2* upstream sequence.

The pGL4.14 empty vector lacking the *TREM2* upstream sequence was used in the luciferase assay. Error bars indicate SD; $n = 3$.

Supplementary Figure S6

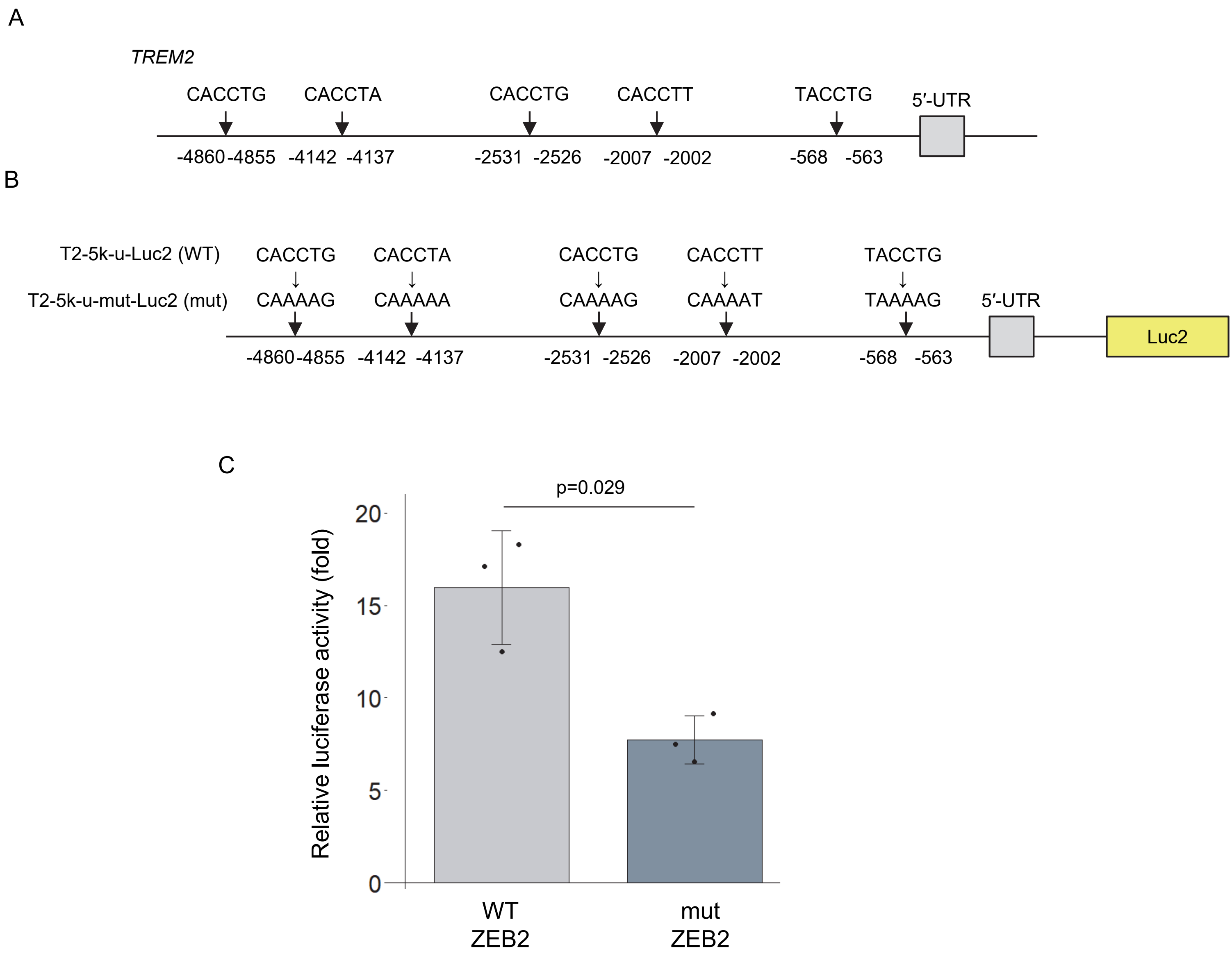

Supplementary Figure S6. **The *TREM2* upstream region contains ZEB2-binding motifs.**

(**A**) The upstream sequence of *TREM2* was predicted to contain two ZEB2-binding motifs (CACCTG) and three additional similar motifs.

(**B**) All five predicted ZEB2-binding motifs were mutated.

(**C**) Quantitative analysis of relative luciferase activity. The relative luciferase activity was normalized to T2-5k-u-Luc2 (WT)-ZEB2. Error bars indicate SD; $n = 3$; Welch's test.

Supplementary Figure S7

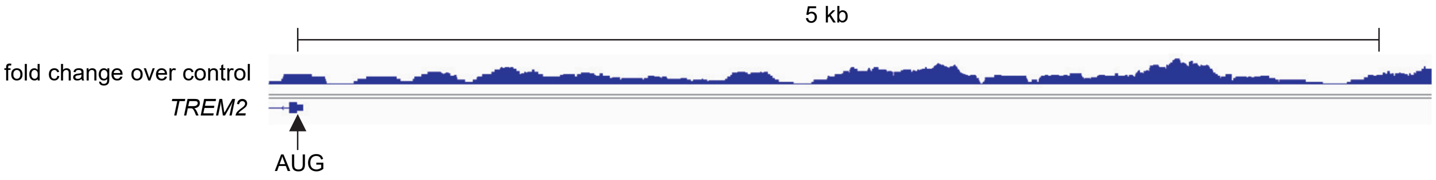

Supplementary Figure S7. ZEB2 ChIP-seq suggests the binding of ZEB2 to the upstream region of *TREM2*. Schematic representation of fold change over control from ZEB2 ChIP-seq reads. ZEB2 ChIP-seq data (ENCSR322CFO) deposited in ENCODE project were visualized using IGV software. ENCSR322CFO was generated in K562 leukemia-derived cells.

Supplementary Figure S8

Fig. 3E

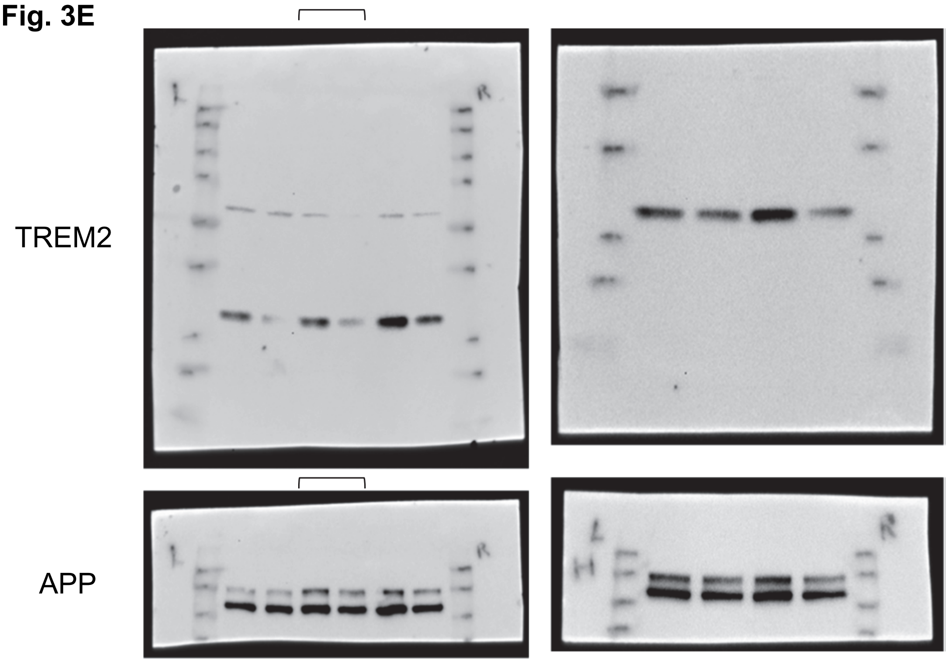

The regions marked in square brackets indicate the portions shown in the figure.
Quantification was performed using the blot data described above.

Fig. S2

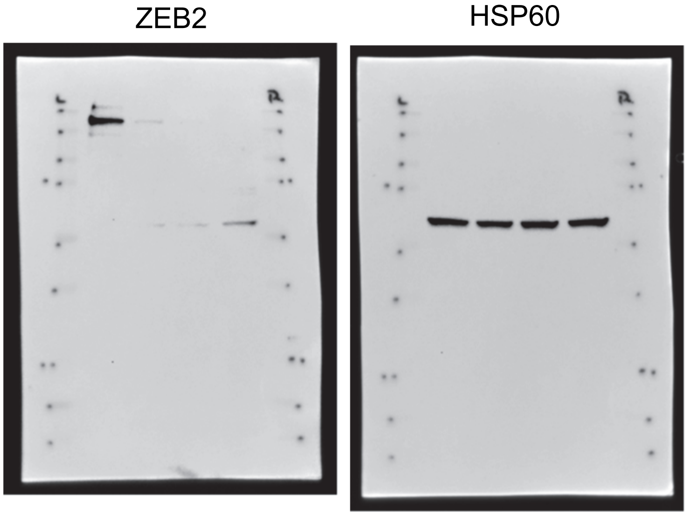

Fig. S3

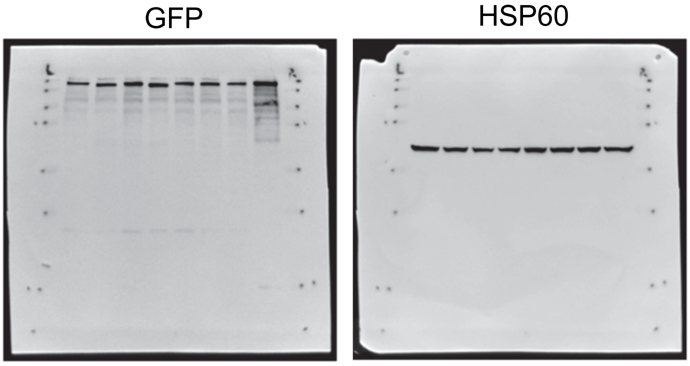

Supplementary Figure S8. Original uncropped images
